# Technologies for the Production of Meat Products with a Low Sodium Chloride Content and Improved Quality Characteristics—A Review

**DOI:** 10.3390/foods10050957

**Published:** 2021-04-28

**Authors:** Tae-Kyung Kim, Hae-In Yong, Samooel Jung, Hyun-Wook Kim, Yun-Sang Choi

**Affiliations:** 1 Research Group of Food Processing, Korea Food Research Institute, Wanju 55365, Korea; privacykin@naver.com (T.-K.K.); awsm_y@kfri.re.kr (H.-I.Y.); 2 Division of Animal and Dairy Science, Chungnam National University, Daejeon 34134, Korea; samooel@cnu.ac.kr; 3 Department of Animal Science & Biotechnology, Gyeongnam National University of Science and Technology, Jinju 52725, Korea

**Keywords:** salt, shelf life, water-holding capacity, emulsion stability, salt reduction

## Abstract

In recent years, consumer concerns regarding high levels of sodium chloride (NaCl) intake have increased, given the associated risk of cardiovascular disease. This has led food industries to consider lowering the use of sodium in food products. However, it is well known that the addition of NaCl to meat products enhances their quality, including water-holding capacity, emulsification capacity, juiciness, and texture. Thus, it is difficult to completely remove salt from meat products; however, it is possible to reduce the salt content using salt substitutes, flavor enhancers, textural enhancers, or other processing technologies. Several recent studies have also suggested that processing technologies, including hot-boning, high pressure, radiation, and pulsed electric fields, can be used to manufacture meat products with reduced salt content. In conclusion, as the complete removal of NaCl from food products is not possible, combined technologies can be used to reduce the NaCl content of meat products, and the appropriate technology should be chosen and studied according to its effects on the quality of the specific meat product.

## 1. Introduction

### 1.1. Role of Sodium Chloride in Meat Products

Sodium chloride (NaCl), commonly known as salt, has been utilized as a food preservative since ancient times [1]. In meat processing, NaCl is an essential additive, and salt has been added from the 1.1 g of salt/100 g in sausages to the 4.6 g of salt/100 g in salami [2]. The addition of NaCl to the cutting process of meat products elutes myofibrillar proteins from meat [2]. The extracted myofibrillar proteins contribute to the emulsion stability of meat by enclosing the fat component and preventing the release of moisture, thereby conferring the desired characteristics to the final product [3]. In other words, the myofibrillar proteins eluted by NaCl bind meat, water, and fat together to give the product the desired texture [4,5]. Adding NaCl to processed meat products can improve texture and maintain a stable form of meat emulsion [3]. NaCl is also known to enhance flavor and juiciness. An increase in juiciness is induced by the extraction of salt-soluble myofibrillar proteins to compensate for the stable state of the proteins bound to water [6]. Finally, NaCl inhibits the growth of microorganisms during the storage of meat products by regulating water activity, osmotic shock, and electrolyte imbalance [7,8,9]. Thus, salt is commonly added during the manufacturing of processed meat products [10].

### 1.2. Risk of NaCl Intake to Health

Salt is an essential component for maintaining human life, as its intake maintains a proper water balance in the body by constantly adjusting fluid and blood levels [11]. For example, salt regulates blood pressure and the excitability of nerves to maintain important physiological functions, such as muscle contraction and the transport of nutrients. However, high sodium intake is associated with an increased risk of developing chronic diseases, such as hypertension, stroke, and cardiovascular disease [3]. The World Health Organization recommends that sodium intake should not exceed 2 g per day and 5 g of salt [11]. Currently, there is a dietary focus on reducing salt intake through consumer education, cooperation between governments and industries, and regulations to prevent chronic diseases and promote good health [12]. People intake sodium through various food sources such as snacks, bread, drinks, and meat, with meat products contributing over 20% of sodium to the diet [2]. Therefore, food manufacturers are exploring novel methods to minimize the addition of salt to processed meat products. In developed countries, suggestions and recommendations for food intake have been developed based on the results of scientific research. The global trend of well-being and the concept of “Lifestyles of Health and Sustainability” have introduced changes in the formulation of additives for food processing. In addition, the UK Food Standard Agency has proposed targets of sodium and equivalent salt level in meat products since 2006 [2]. Despite all this, the proper salt content is essential to maintaining the quality of meat products.

### 1.3. Purpose of this Study

This study aims to compare newer alternatives to the standard methods of improving the physicochemical properties (such as water holding capacity, texture, and antimicrobial properties) of reduced-salt meat products [13], and these should also maintain the palatability of meat and meat products with reducing salt addition [14]. Thus, in this paper, there are several alternative processing methods leading to the production of meat products with reduced salt content while maintaining the palatability and the microbial stability of the products. Overall, this review aims to provide an overview of the traditional and advanced technologies for the development of low-salt meat products. In particular, we consider innovative manufacturing technologies for the production of low-salt processed meat that confer physicochemical properties, shelf life, and palatability similar to those of the existing processes.

## 2. Alternative Processing Techniques

The meat industry has consistently attempted to apply technical approaches to improve the quality attributes of low-salt meat products, including technologies that help prevent the deterioration of texture and microbial spoilage. While there is as yet no perfect processing solution to comprehensively improve the overall quality of low-salt meat products, it is worth noting that there is potential to improve the quality defects of low-salt meat products. In this section, the modern processing technologies that can be used to improve the stability, quality, and shelf life of low-salt meat products are introduced and discussed (Table 1).

### 2.1. Hot-Boning Technology

Skeletal muscles undergo several metabolic processes after slaughter—mainly due to the depletion of biological energy sources (e.g., adenosine triphosphate (ATP) and creatine phosphate) and the disruption of oxygen supply—in which the muscle pH decreases with anaerobic postmortem glycolysis [15]. As a result, rigor mortis occurs, which causes a decline in the major processing characteristics such as protein solubility and water-holding capacity [16]. In this regard, a hot-boning technique employed to obtain pre-rigor muscles that have excellent processing characteristics with high pH and relaxed microstructure has been proposed, not only to improve the quality attributes of the final products but also to reduce economic losses during storage and distribution [17]. However, in many countries, there is a limitation on the production of pre-rigor meat since it requires appropriate facilities that guarantee a continuous process of slaughter, sectioning, deboning, and processing before rigor mortis begins. In addition, because of the high temperature, microbial contamination, and higher microbe growth rate involved in this process compared to conventional ones, this process should be regulated [18]. Because salting and batter formulation has a higher impact on the microbial growth of meat products than deboning time, pre-rigor salting is an important process when use hot-boning technology [19].

The concept of using pre-rigor meat to improve the characteristics of low-salt meat products has been described by Desmond [2], and previous studies have determined the technical properties and oxidative stability of pre-rigor muscle at low salt concentrations [15,16]. Previous studies explored pre-rigor salting effects and found that the addition of at least 2% NaCl was necessary to guarantee an improvement in meat characteristics such as protein solubility, emulsifying capacity, water-holding capacity, and regulation of microbial growth, which further facilitated the inactivation of glycolytic enzymes [3,16,19]. In this regard, the addition of a minimum amount of salt to inhibit postmortem anaerobic glycolysis can limit the use of pre-rigor salted meat in low-salt meat products. As an alternative strategy, Choi et al. [3] prepared a mixture of pre-rigor salted and post-rigor salted meat in a meat emulsion at a reduced salt concentration (1% NaCl). Furthermore, the salt concentration in the pre-rigor salting process also had a significant effect on the quality of the final meat product. When pre-rigor salt concentrations from 1% to 5% were compared, the salt concentration of 3% was the ideal addition amount [3]. Since potassium chloride (KCl) is commonly used as a salt substitute, Song et al. [20] recently evaluated the effect of pre-rigor salting with KCl on the technical properties of meat and suggested that KCl had a milder effect on meat than NaCl. Pre-rigor salting with KCl improved water holding capacity, pH value, and protein solubility when compared with post-rigor salting with NaCl.

### 2.2. High-Pressure Processing

High-pressure processing is primarily considered an effective non-thermal preservation method in the meat industry, which can be used to extend the shelf stability of low-salt meat products by inactivating spoilage-inducing microorganisms [21]. In addition, previous studies have found positive effects of high-pressure processing on the technical properties and microbial safety of meat products. When high-pressure processing is used, the functional properties of protein molecules can be altered, and the enzymatic activity of meat can be modulated [22]. Owing to these properties, several investigators have attempted to manufacture low-salt meat products using this process.

O’Flynn et al. [5] found that the use of high-pressure-treated raw meat (at 150 MPa) reduced cooking loss in breakfast sausages, irrespective of the salt concentration, and suggested that high-pressure-treated meat could be useful in producing low-salt sausages containing 1.5% NaCl. Zheng et al. [23] reported that high-pressure treatment (0.1, 200, and 400 MPa) could improve the physicochemical properties of low-salt meat batters, depending on the intensity of the applied pressure, and suggested that the use of a lower pressure intensity (200 MPa) could be beneficial for the production of low-salt meat products. Moreover, Tamm et al. [6] indicated that the application of high pressure (100 MPa) using 0.2% KCl, a salt replacer, could be a practical strategy to produce reduced-salt cooked ham.

NaCl can be replaced by high-pressure treatment because such treatment can extract myofibrillar proteins from muscles, which is one of the roles of salt. According to Lee et al. [24], the high-pressure treatment (200 MPa) of meat batter leads to an increase in the solubility of myofibrillar proteins, including myosin and actin. Similar results were also reported by Iwasaki et al. [22]. When subjected to sodium dodecyl sulfate–polyacrylamide gel electrophoresis, the band densities of myosin heavy chain, α-actinin, actin, and tropomyosin were observed to be increased in meat samples treated at a high pressure of 200 MPa, whereas they were found to be decreased in samples treated at 100 and 300 MPa. This result indicates that it is difficult to replace salt in meat products using high pressure when the treatment exceeds 300 MPa. Likewise, several studies have reported that an excessive intensity of high-pressure treatment could potentially have negative impacts on the technical properties of raw meat [6], and that such treatment could negatively affect the physicochemical properties of low-salt meat products. Thus, the addition of salt replacers or functional ingredients together with an appropriate high-pressure treatment (100–200 MPa) to improve the physicochemical and microbiological properties of meat products may be considered for low-salt food manufacturing applications. However, the expensive initial investment and high operating and maintenance costs lead to increased meat product prices, and the consumer opinion of new technologies could be an obstacle when applying high-pressure processing. Therefore, hurdle technology should be developed to increase safety and moderate costs [25].

### 2.3. Radiation

Food irradiation technology is used as a non-thermal preservation method in the food industry for ripening and the control of microbial growth. In general, three ionizing sources (gamma rays, electron beams, and X-rays) are used for food irradiation, and their effectiveness in extending the shelf life of fresh meat and processed meat products has been documented and well-reviewed [26]. However, an accelerated deterioration in physicochemical properties has been reported, induced mainly by oxidative stress, such as discoloration and the oxidation of biological molecules, including lipids and proteins, and the formation of irradiation off-flavors and odors can occur in irradiated meat products [27,28].

The food irradiation technique, with its excellent sterilization effect, has been used in several low-salt foods that may be vulnerable to spoilage due to microbial growth [13]. Particularly in processed meat products with relatively low salt concentrations (around 2%), gamma-ray treatment at 2 kGy was found to reduce aerobic mesophilic microbial counts from 7.6 to 1.2 log_10_ counts/g under 7 °C storage conditions for 1 week. Recently, Song et al. [13] reported that the three ionizing sources (gamma rays, electron beams, and X-rays) at 5 kGy had a positive impact on the microbial safety (absence of aerobic microbes, coliforms and Enterobacteriaceae, and Pseudomonas spp.) of low-salt emulsion sausages (0.75% NaCl) during 4 weeks of chilled storage. Thus, it is practically possible to ensure the microbiological safety of low-salt sausages through food irradiation techniques. Although microbial growth could be controlled efficiently, accelerated oxidation could be induced by irradiation and the deterioration of meat products could be accelerated [29]. In addition, negative consumer acceptance of irradiated foods and the high costs of operating and maintenance are still critical problems that need to be considered.

### 2.4. Ultrasound

Power ultrasound has been considered in the meat industry for use during processing phases, as it contributes to improving the technical properties of meat. This technology has been used commercially because of its high speed, reliability, low cost, and simple application [30]. In general, two types of ultrasound—high-frequency (>1 MHz) and low-intensity (<1 W cm^-2^), and low-frequency (20–100 kHz) and high-intensity (10–1000 W cm^-2^)—have generally been used in previous studies [31]. In previous studies, the major effects of ultrasound on the technical properties of meat during processing have been reported to be as follows: accelerated tenderization [32], improved water-holding capacity [33], increased efficacy of salt diffusion [34], and reduced microbial growth [35]. With respect to the development of low-salt meat products, the positive effects of ultrasound on water-holding capacity and microbial safety are worthy of attention. In fact, previous studies have evaluated the possibility of using ultrasound to improve the quality attributes of low-salt meat products. Li et al. [36] found that high-power ultrasound treatment (40 kHz with 300 W) improved the gelation properties of meat batter, though significant results for water-holding capacity and texture were not obtained. Recently, Barretto et al. [37] reported that high-intensity ultrasound (600 W) treatment decreased total fluid release but increased the hardness of restructured cooked ham containing 0.75% salt, resulting in improved sensory acceptance. Although there has been little to no information on the impact of ultrasound on the growth of spoilage-causing or pathogenic bacteria in meat products with different salt concentrations, it has been suggested that the antimicrobial effect of ultrasound could be associated with the production of hydrogen peroxide and free radicals [38]. In further studies, if the antimicrobial effects of ultrasound on processed meat products with different salt concentrations are investigated and proven, then this method could be leveraged as a useful technique for improving the technical properties and microbial safety of low-salt meat products.

### 2.5. Pulsed Electric Field Processing

Pulsed electric field processing is a non-thermal technology mainly used in food processing to improve food quality and extend shelf life [39]. The primary advantage of pulsed electric field technology is that it increases the microbiological safety of processed food without considerably affecting its nutritional and sensory characteristics [40]. In addition, the application of pulsed electric field technology in food processing has the advantages of low-temperature treatment, short processing time, and the potential for continuous flow; electric field pulses of very short duration and high intensity are applied to food placed between electrodes with an electric field strength of 0.3–80 kV/cm [41]. While the first commercial application of electric field pulses in food processing was to preserve fruit juice, the technology could benefit many other processes such as cold pasteurization and sterilization, food dehydration, freezing processes, food product allergenicity reduction, enzyme inactivation, and the extraction and recovery of bioactive compounds [41].

Previous studies have been conducted on fresh meat and meat products and reported the effect of pulsed electric fields, mainly on the tenderness and other quality parameters of different animal muscles [39,40,41]. Toepfl et al. [42] indicated that appropriate meat tenderness could be accomplished using an electric field strength of 1–10 kV/cm and an energy input of 0.5–10 kJ/kg. They also revealed the mechanism of action of the pulsed electric field, showing that the tenderization benefit of the pulsed electric field was achieved through increased proteolysis [43]. Research on utilizing pulsed electric field processing for low-salt meat products is limited. Bhat et al. [44] conducted a study to investigate whether pulsed electric field processing is an effective approach for salt reduction in meat products. This study showed that the pulsed electric field processing of beef jerky permitted direct salt reduction without any damaging effects on the lipid oxidation, sensory quality, and the microbial stability of the product. The pulsed electric field improved salt diffusion and distribution in the meat matrix and perception during chewing, as well as possibly sodium release. The pulsed electric field processing of the meat did not influence color or cooking loss, and the tenderness of the end-products was found to be excellent. Thus, the technology of pulsed electric field processing warrants further studies for beneficial applications in the manufacturing of low-salt meat products in the future.

**Table 1:** Studies regarding the alternative processing techniques for salt reduction in meat products.

Processing Technology	Product Category and Detailed Method	Significant Effects	Reference
Hot-boning technology	Chicken breast Mixed addition of pre-rigor salted chicken breast with cold-boned chicken breast	Reduced cooking loss Improved emulsion stability	[3]
	Chicken breast Pre-rigor chicken breast salted with KCl	Increased redness Decreased hardness, gumminess, and chewiness Low overall sensory	[20]
High-pressure processing	Breakfast sausage Raw meat treated with high pressure for producing low-salt breakfast sausages (1.5% NaCl)	Reduced cooking loss Increased emulsion stability	[5]
	Chicken meat batter Optimization of high-pressure conditions for improving technological properties	Increased water holding capacity and hardness, and sensory properties (best results for physicochemical properties obtained at 200 MPa among 0.1, 200, and 400 MPa)	[23]
	Cooked ham Combined application of high pressure and 0.2% salt replacer (KCl) in reduced-salt cooked ham	The production of reduced-salt cooked ham without adverse impacts on water binding and texture in comparison to the product with 1.9% NaCl	[6]
Radiation	Emulsion sausage Ionizing irradiation on low-salt emulsion sausage (0.75% NaCl)	Inhibition of the growth of aerobic microbes, coliforms, *Enterobacteriaceae*, and *Pseudomonas* spp. during chilled storage	[13]
Ultrasound	Chicken breast meat batter	Improved gel properties	[36]
	Restructured cooked ham with 0.75% salt	Decreased total fluid release and increased hardness	[37]
Pulsed electric field	Beef jerky Pulsed electric field processing in low-salt beef jerky (1.2% NaCl)	Improved the salt diffusion and distribution in the meat matrix and improved the saltiness naturally	[41]
	Loin Deer *Longissimus dorsi* in pulsed electric field	Higher soluble protein and digestibility of muscle	[44]

## 3. Salt Substitutes in Meat Products

Low-salt meat products have a low ability to retain moisture during the manufacturing process, as the extractability of salt-soluble proteins decreases. Hence, during the manufacturing process of low-salt meat products, it is necessary to introduce new substances to replace the salt-soluble proteins that can induce water retention and desirable physical properties. Table 2 shows the salt substitutes used for the treatment of meat products.

### 3.1. Metallic Agents

The most important factor for replacing salt in prepared meat products is the use of alternative substances that can effectively substitute the beneficial effects of salt. When the effects of different sodium anion species on turkey breast meat were compared, the partial replacement of NaCl with Na_2_HPO_4_ could reduce sodium content by 20% [45]. KCl has demonstrated comparable antimicrobial effects to NaCl on pathogenic bacteria in laboratory media [7]. The microorganism counts in sausages were similar even when the NaCl concentration was reduced to 1%, and the reduction of NaCl in meat products had no effect on the growth of Pseudomonas, Enterobacteriaceae, and Brochothrix [46]. Although microbial growth was not affected when NaCl was replaced by other metallic salts, the duration of post-salting had to be increased to reach an appropriate level of water activity when manufacturing dry-cured meat [47]. Such metallic salts have also been used to develop starter cultures in the manufacture of low-salt dry-fermented sausages [48]. Low-salt emulsified sausages did not show a significant difference in microbial growth when compared with traditional formula sausages, even when more than half of the NaCl concentration was replaced with KCl and calcium chloride [49]. An approximately 25% reduction in NaCl content or 18% reduction in sodium content was found to be practical for the manufacture of frankfurters [50]. The sodium content of 0.6% NaCl could be replaced by 1.2% sodium lactate; the addition of 1% sodium lactate did not exhibit a specific difference in microbial growth compared with that in the original meat products [50]. The aforementioned metallic salts are thus considered to be useful replacements for NaCl for the inhibition of microbial growth in processed meat. However, KCl has critical disadvantages in terms of the textural properties and flavor of meat products, resulting in a salty taste [51]. For these reasons, only 50% NaCl could be replaced by KCl when manufacturing emulsified meat products due to factors related to the sensory physicochemical properties of the meat [52]. Salt mixtures containing KCl, CaCl_2_, or MgCl_2_ ensure proper texture; however, their major disadvantage related to the flavor of meat products can be an obstacle. The unsavory flavors of these alternative salts could be mitigated by partially reducing their content using NaCl. NaCl (50%) could be replaced by a mixture of KCl and CaCl_2_ in jerked beef, and KCl was found to be the best salt substitute [53]. Furthermore, the use of potassium can potentially cause certain diseases in some vulnerable populations; therefore, an intake of 4.7 g of potassium per day is recommended [2]. Because of these disadvantages, other ingredients have been studied to enhance the flavor and textural properties of meat products.

### 3.2. Natural Enhancement

In addition to metallic salts, other ingredients, such as yeast extracts, essential oils, lactates, monosodium glutamate, liquid smoke, and nucleotides, are also added to compensate for the disadvantages of salt reduction to enhance flavor and improve the texture of meat products [54,55]. As described below, many manufacturers have developed salt reducers, salt replacers, and flavor enhancers combined with/without metallic salts. Fermented red beet extract is not only used to control the growth of microorganisms but also to enhance flavor [56]. Savory powder is used to enhance the flavor of meat products and mask undesirable flavors [57]. Brewed soy sauce and fermented flavor enhancers have also been studied as flavor enhancers for low-salt bacon, beef jerky, summer sausages, and boneless ham [58]. Yeast extracts (lactates, monosodium glutamate, amino acids, and nucleotides), plant extracts (polyphenols and essential oils), and the smoking process are also used for the enhancement of flavor [21]. Yeast extracts such as monosodium glutamate, disodium inosinate, disodium guanylate, lysine, and taurine improved the sensory properties of reduced-salt, fermented, cooked sausages [59]. Plant extracts are also used as a good additive to improve shelf life and health-promoting attributes [60]. Anthocyanin, tannin, and flavonoids are considered as high antioxidant agents, and these components are abundant in natural plants [60]. Essential oils obtained from plants such as *Lamiaceae*, *Lauraceae*, *Myristiceae*, *Myrtaceae*, *Umbelliferae/Apiaceae*, and *Zingiberaceae* families have applications as antimicrobial agents [60,61]. To improve the activation of these components, active films and encapsulation processing are also applied [61]. Changes in the shape of salt crystals are other methods of reducing salt content, as different ion diffusion levels and the rapid action of the modified salt in a meat network could lead to an enhancement of the salty taste [62]. Because NaCl plays an important role in retaining the textural properties of meat products, alternative ways to improve the textural properties must be undertaken when reducing the concentration of NaCl. Hydrocolloids, such as binding proteins, polysaccharides, and enzymatic reactions, can be used to enhance the textural properties of low-salt meat products [63]. A combination of fibrin and thrombin with microbial transglutaminase can replace the NaCl content of beef patties [64]. Altering the size of NaCl crystals could also be a means of reducing regular salt content; approximately 33% of salt in beef patties could be reduced when using micronized salt [65]. Polyphosphates can also be used to enhance the textural properties of meat by increasing the pH of meat products [21].

**Table 2:** Studies regarding the salt substitutes used for salt reduction in meat products.

Product Category	Reduced or Replaced Sodium Amount (%)	Sodium Chloride Substitutes	Reference
*Ground meat*			
Mortadella	50–75 (50) ^1)^	CaCl_2_, MgCl_2_, KCl	[52]
Frankfurter	33 (33)	Fermented red beet	[56]
Dry-fermented sausage	61 (61)	KCl, CaCl_2_	[48]
Bologna	20, 40, and 60 (40)	PuraQ^®^ Arome Na4	[14]
	50 (50)	Lysine, liquid smoke, KCl	[55]
Deli type sausage	25–50 (50)	Soda-Lo^®^ salt	[62]
Smoked sausage	45–50 (50)	OF-45LSN, OF-60LSN, Savory powder	[57]
Restructured ham	40–45 (45)	OF-45LSN, OF-60LSN, Savory powder	[57]
Summer sausage	30–50	KCl, soy sauce, fermented flavor enhancer	[58]
Black pudding	66.66	Wheat bran, sodium citrate, carrageenan, pectin, KCl, glycine, carboxymethylcellulose, seaweed wakame, PuraQ^®^Aroma NA4, KPO_4_, waxy maize starch	[63]
Ground beef meat	50–75	Microbial transglutaminase, fibrimex, alginate	[64]
*Whole muscle*			
Beef jerky	50 (50)	KCl, CaCl_2_	[53]
	30–50 (30)	KCl, soy sauce, fermented flavor enhancer	[58]
Cooked ham	25–50 (50)	Soda-Lo^®^ salt	[62]
Turkey breast	25–50 (50)	Soda-Lo^®^ salt	[62]
	20–46 (20)	Na_2_HPO_4,_ Na_5_P_3_O_10_, Na_2_SO_4_, C_5_H_8_NNaO_4_,···	[45]
Dry-cured ham	50–55 (55)	CaCl_2_, MgCl_2_, KCl	[47]
Bacon	30–50 (50)	KCl, soy sauce, fermented flavor enhancer	[58]
Boneless ham	30–50 (30)	KCl, soy sauce, fermented flavor enhancer	[52]

^1)^ Numbers in parentheses indicate the maximum reduced level (%) without significant differences from regular products.

## 4. Conclusions

The aim of this review was to summarize the approaches adopted in traditional and advanced processing technologies for producing low-salt meat products. Alternative substances and processing technologies for reducing or replacing sodium in meat products are being actively researched with the goal of the industrial development and manufacture of low-salt meat products. Studies have shown that NaCl cannot be replaced or reduced by over 50% using a single process. Therefore, in order to ensure the processing suitability, flavor, and microbiological safety of processed meat products with a low salt content, a combination treatment technology has to be applied rather than the use of a single substitute technology, thus necessitating more research on such combined approaches. Parameters such as curing efficiency, shelf life, and texture have a significant impact on the choice of the applied salt reduction method.

## Data Availability

Not applicable.
